# Foetal Immune Response Activation and High Replication Rate during Generation of Classical Swine Fever Congenital Infection

**DOI:** 10.3390/pathogens9040285

**Published:** 2020-04-14

**Authors:** José Alejandro Bohórquez, Sara Muñoz-González, Marta Pérez-Simó, Iván Muñoz, Rosa Rosell, Liani Coronado, Mariano Domingo, Llilianne Ganges

**Affiliations:** 1 OIE Reference Laboratory for Classical Swine Fever, IRTA-CReSA, 08193 Barcelona, Spain; josealejandro.bohorquez@irta.cat (J.A.B.); sara.vets.11@gmail.com (S.M.-G.); marta.perez@irta.cat (M.P.-S.); ivan.munoz@irta.cat (I.M.); rosa.rosell@irta.cat (R.R.); lianicoronado@gmail.com (L.C.); mariano.domingo@uab.cat (M.D.); 2 Departament d’Agricultura, Ramadería, Pesca, Alimentació I Medi Natural i Rural (DAAM), 08007 Generalitat de Catalunya, Spain; 3 Centro Nacional de Sanidad Agropecuaria (CENSA), Mayabeque 32700, Cuba; 4 Servei de Diagnòstic de Patologia Veterinària (SDPV), Departament de Sanitat I d’Anatomia Animals, Universitat Autònoma de Barcelona, Bellaterra, 08193 Barcelona, Spain

**Keywords:** classical swine fever, virulence, trans-placental transmission, persistent congenital infection, foetal immune response, classical swine fever virus, replication, sows

## Abstract

Classical swine fever virus (CSFV) induces trans-placental transmission and congenital viral persistence; however, the available information is not updated. Three groups of sows were infected at mid-gestation with either a high, moderate or low virulence CSFV strains. Foetuses from sows infected with high or low virulence strain were obtained before delivery and piglets from sows infected with the moderate virulence strain were studied for 32 days after birth. The low virulence strain generated lower CSFV RNA load and the lowest proportion of trans-placental transmission. Severe lesions and mummifications were observed in foetuses infected with the high virulence strain. Sows infected with the moderately virulence strain showed stillbirths and mummifications, one of them delivered live piglets, all CSFV persistently infected. Efficient trans-placental transmission was detected in sows infected with the high and moderate virulence strain. The trans-placental transmission occurred before the onset of antibody response, which started at 14 days after infection in these sows and was influenced by replication efficacy of the infecting strain. Fast and solid immunity after sow vaccination is required for prevention of congenital viral persistence. An increase in the CD8+ T-cell subset and IFN-alpha response was found in viremic foetuses, or in those that showed higher viral replication in tissue, showing the CSFV recognition capacity by the foetal immune system after trans-placental infection.

## 1. Introduction

Classical swine fever virus (CSFV) is one of the most relevant viruses in the Pestivirus genus, being the causative agent of classical swine fever (CSF), a highly impactful disease for the porcine industry worldwide [1]. The capacity of pestiviruses to generate persistent infection by trans-placental transmission has already been described [2,3,4,5,6]. Particularly, low virulence CSFV strains have been related to the development of congenital viral persistence in their offspring when infection of the sows occurs between 50 and 90 days of gestation [1,2,3,4,5]. Piglets that develop this form of infection are born infected, showing high viral replication and shedding in the absence of specific antibody response [3,4,7]. This type of viral persistence has been explained by the immunotolerance mechanism, due to a lack of CSFV recognition by the immature immune system of the foetus [5]. 

CSF still remains endemic in countries in Asia, the Caribbean, and Central and South America [1]. Previous studies have demonstrated the evolutionary capacity of CSFV towards less virulent strains in endemic situations under inefficient vaccination programs [8,9]. In this type of scenario, a recent study showed that CSF persistence was the predominant form, favoring virus prevalence and hampering the control tools [10].

CSFV also has the ability to generate viral persistence after postnatal infection, although unlike the congenital persistence forms, the generation of postnatal persistence has been associated with the CSFV moderate virulence strains [11,12]. Previous studies have also shown that moderate virulence strains are widely distributed [13,14,15]. In this regard, the strain of CSFV that recently caused an epidemic in Japan after 26 years has been characterised to be of moderate virulence [16,17]. 

Despite the known capacity of CSFV to be transmitted by the trans-placental route and to induce persistent congenital infection, few scientific works have dealt with the immunopathogenesis of this form of the disease, especially from a virus–host interaction standpoint. Considering this background, the aim of this work is to evaluate the capacity of CSFV strains with different virulence degrees to infect pregnant sows and its relation with the vertical transmission by trans-placental infection of fetuses. Likewise, the implication of the virulence degree in the generation of CSFV congenital persistent infection is also assessed. The levels of viral replication, as well as the immune response, in terms of cytokine production and changes in immune system cell populations were evaluated in foetuses and piglets from the infected sows.

## 2. Results

### 2.1. Clinical Evaluation of Sows Infected with Pinar del Rio (PdR) vs. Margarita CSFV Strains 

In the first experiment, aiming to determine the capacity of CSFV strains of different virulence levels to induce trans-placental infection, two groups of pregnant sows were inoculated with CSFV at 74 days of gestation. Group A (Sows 1 and 2) was infected with the highly virulent CSFV Margarita strain, while Group B (Sows 3 and 4) were inoculated with the low virulence PdR strain. Clinical signs were recorded daily by a trained veterinarian in a blinded manner.

After inoculation, both CSFV Margarita-infected sows (Group A) showed anorexia and apathy between 6 and 11 days post-infection (dpi). Subsequently, Sow 2 started to eat normally, whereas the clinical condition of Sow 1 deteriorated progressively, showing constipation/diarrhoea, some peaks of fever, evident weight loss, and, eventually, weakness of the hindquarters. This animal was euthanised at 17 dpi (91 days of gestation) for animal welfare reasons, while the remaining sows were euthanised at 22 dpi (96 days of gestation). Both Margarita infected sows showed similar lesions at necropsy, consisting of petechiae in the kidneys, stomach, and intestine, and, in the case of Sow 1, also in the urinary bladder. Conversely, Sows 3 and 4, inoculated with the PdR strain, remained healthy throughout the study, and no lesions related to CSFV infection were found at necropsy. 

### 2.2. CSFV RNA Level Detected in Sows after Infection with Margarita or PdR Strains

**Figure 1:**
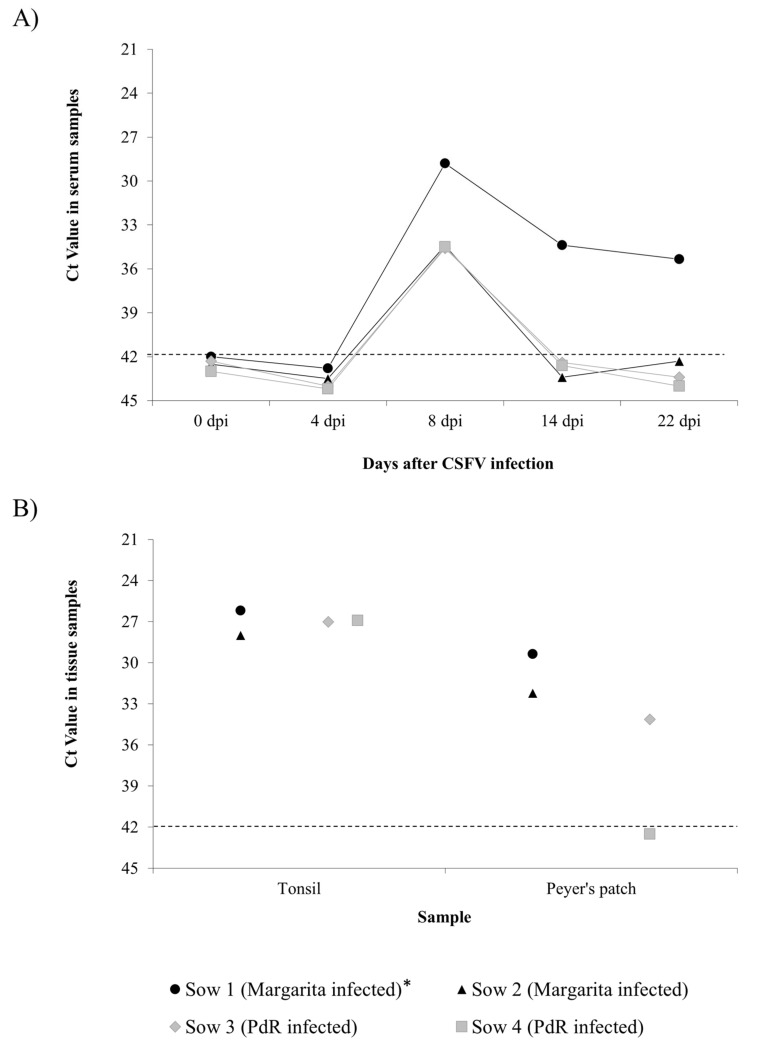
Classical swine fever virus (CSFV) RNA detection by RT-qPCR in sow samples. (**A**) RNA levels detected in sera at different times post-infection. (**B**) RNA levels detected in tissues from sows infected with either the CSFV Margarita (black symbols) or PdR strain (grey symbols). Cycle threshold (Ct) values over 42 (dotted line) were considered as negative. Asterisk indicates the animal that was euthanised at 17 dpi.

CSFV RNA was evaluated by reverse transcription-quantitative PCR (RT-qPCR) [18] in serum samples collected weekly and tissue samples collected at necropsy. The RNA load was characterised as high, moderate or low in accordance with the cycle threshold (Ct) value, as described in the materials and methods section. The CSFV RNA load detected in sera oscillated from moderate to low load (Ct value from 28 to 35) regardless of the virulence degree of the strain used to infect the sows. The RNA was detected at 8 dpi in all the animals in Group A and B (infected with Margarita or PdR strains, respectively). However, at 14 dpi, and until the end of the experiment, samples from the two animals in Group B and from Sow 2 (Group A) were negative (Figure 1A). Notably, only Sow 1 infected with the CSFV Margarita strain was positive at 14 dpi and at the time of euthanasia (17 dpi), although with low RNA load (Ct value 34).

In the tissue samples, the CSFV RNA load detected in the tonsils samples was similar in both experimental groups, with Ct value around 27 (moderate RNA load). The viral RNA load in Peyer’s patch samples was also similar for both groups, with the exception of Sow 4 (PdR infected), which was negative (Figure 1B). 

### 2.3. The High Virulence CSFV Strain Margarita Elicited Faster and Higher Humoral Response than PdR Strain in the Infected Sows

**Figure 2:**
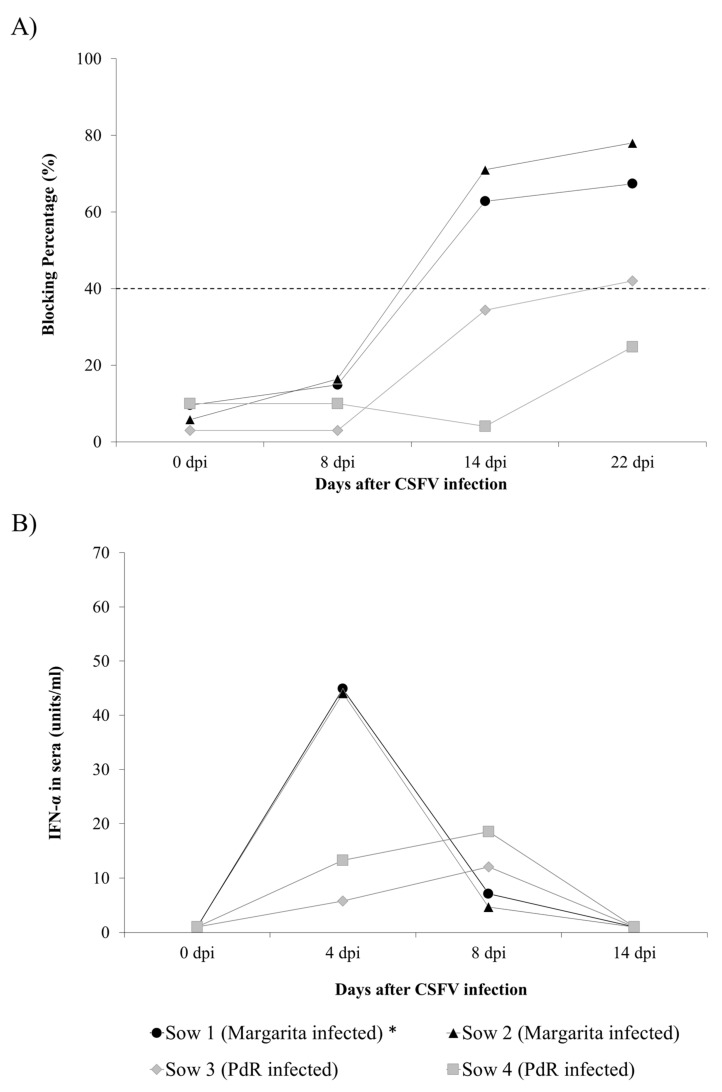
Immune response of sows after CSFV infection. (**A**) CSFV specific anti-E2 antibody response against the E2 glycoprotein detected by ELISA (in blocking %), values above 40% (dotted line) being considered as positive. (**B**) Interferon alpha (IFN-α) response in serum determined by ELISA test from sows infected with either the CSFV Margarita (black symbols) or PdR strain (grey symbols). The IFN-α concentration in sera is expressed as units/mL. Asterisk indicates the animal that was euthanised at 17 dpi.

Specific anti-E2 and neutralising antibodies were evaluated weekly in sera by ELISA and neutralisation peroxidase linked assay (NPLA) [19], respectively. Anti-E2 antibodies were detected in both of the CSFV Margarita-infected sows (Group A) at 14 dpi and at the time of euthanasia. In Group B, infected with the PdR strain, only one animal showed anti-E2 antibodies at 22 dpi (Figure 2A). 

Similarly, both of the Margarita-infected sows showed neutralising antibody titers by NPLA assay starting at 14 dpi, which increased at 17 and 22 dpi for Sows 1 and 2, respectively. In the case of PdR infected sows, neutralising antibody response was only detected in Sow 3 at 22dpi, while sow 4 did not show neutralising antibodies throughout the whole trial (Table 1).

### 2.4. IFN-α and IFN-γ Response in Sows Infected with High or Low Virulence CSFV Strains 

Interferon alpha (IFN-α) and interferon gamma (IFN-γ) were evaluated by ELISA test in sera from sows at different time-points after infection. IFN-α was detected in the sera of all the sows from Groups A and B at 4 and 8 dpi. Notably, the highest levels were registered in sows from Group A (Sows 1 and 2) at 4 dpi (Figure 2B). For IFN-γ, no detectable levels in sera were found in either experimental group after infection.

### 2.5. Evaluation of the Foetuses from CSFV Infected Sows at Necropsy

Foetuses from the CSFV Margarita-infected sows showed internal haemorrhages in tonsil, intestine, kidneys, lymph nodes and spleen. Furthermore, four of the foetuses from Sow 1 and three foetuses from Sow 2 had generalised haemorrhagic lesions in the skin (data not shown). Additionally, one mummified foetus was found in both of them. Conversely, foetuses from Sows 3 and 4, inoculated with the PdR low virulence strain, showed no lesions at necropsy. 

### 2.6. Vertical Transmission and CSFV Replication in the Foetuses

Following hysterectomy, serum and tissue samples were collected from all the foetuses, about two weeks before the expected delivery day, in order to determine CSFV transmission from sows to their foetuses. All the foetuses from Group A were RT-qPCR positive with high CSFV RNA load (Ct values between 15.67 and 23) in the majority of sera, tonsil, spleen and thymus samples (see Table 2). In the different organs, the mean Ct value ranged from 17.28 to 20.33 for foetuses from Sows 1 and 2, respectively. By contrast, only 3 out of 13 (23%) foetuses from each of the PdR-infected sows were positive in sera by RT-qPCR, ranging from high to low RNA load (Table 2). However, after analysis by RT-qPCR of tonsil, spleen and thymus samples, the number CSFV positive foetuses increased to 11 out of 13 (Sow 3) and 9 out of 13 (Sow 4), respectively, with high to low CSFV RNA load in the positive tissues. 

### 2.7. Immune Response in the Foetuses from CSFV-Infected Sows

**Figure 3:**
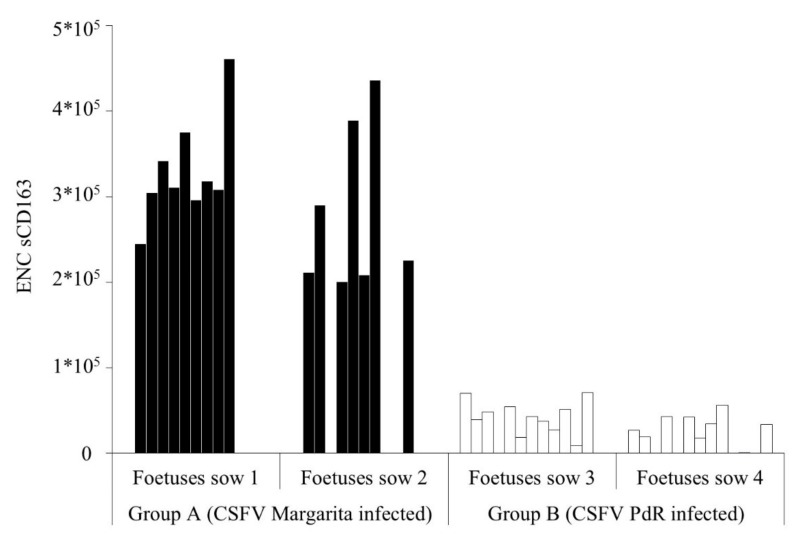
sCD163 levels in foetal sera. Foetuses from sows infected with either the Margarita (black bars) or PdR (white bars) CSFV strains are represented. Results are expressed as the equivalent number of copies of CD163 transfected cells.

Absence of CSFV specific humoral response was found in sera from all the foetuses in the study. However, IFN-γ levels were detected only in serum sample of five out of the 13 foetuses from the Sow 1 (infected with Margarita strain), in values ranging between 23.2 and 130.9 pg/ml. In addition, detectable levels of IFN-α were also registered in 11 samples in foetuses from both Margarita-infected sows (Table 3). Interestingly, foetuses from sows infected with the low virulence strain (PdR) showed higher levels of IFN-α (between 100 to 200 units/ml). Notably, the positive values were found in the foetuses that were CSFV RNA positive for the four samples analysed or in those that showed the higher CSFV RNA load in the tissue samples (Table 2 and Table 3). Finally, detectable levels of soluble CD163 (sCD163) were found in foetal sera samples from both experimental groups, being about 10 times higher the concentration in samples from Group A (Figure 3).

### 2.8. Phenotypical Profile in Foetal PBMCs after CSFV Infection

**Figure 4:**
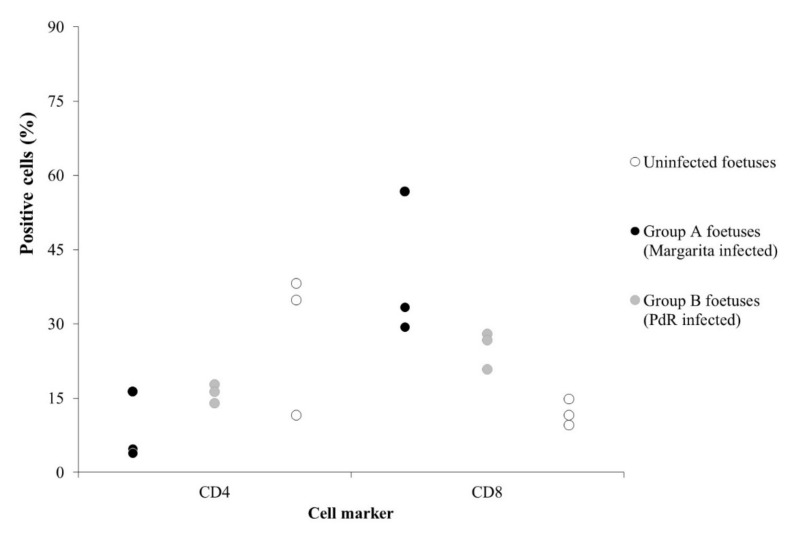
Comparative expression of the CD4+ and CD8+ T-cell subsets in PBMCs from uninfected foetuses (white dots), foetuses infected with the Margarita (black dots), or the PdR (grey dots) CSFV strain.

Samples from whole blood were obtained from three foetuses in each infected group, and peripheral blood mononuclear cells (PBMCs) were isolated. Flow cytometry analysis was performed to study the phenotypical profile in these cells. The PBMCs analysed corresponded with foetuses that showed CSFV RNA levels in serum samples. Additionally, PBMCs of three foetuses from uninfected sows, from the same farm of origin, were also analysed to use as reference, uninfected controls. The CD4^+^ T-cell subset ranged from 4% to 16% of PBMC from the Margarita infected foetuses (Group A), showing a reduction in two out of three samples analysed with values below 5% (Figure 4). This cell population ranged from 14% to 17% in the three foetal PBMC tested from Group B, while a wider range was detected in the PBMC from naïve samples (from 11% to 38%). By contrast, the CD8^+^ T-cells were increased in the CSFV infected foetuses, with percentages between 29% and 56% in Group A, and 20% to 27% in Group B (infected with PdR strain), whereas it was always below 15% for the naïve samples (Figure 4).

### 2.9. Infection with the CSFV Moderately Virulent Strain: Clinical Signs and CSFV Replication in Sows 

In the second experiment, in order to evaluate the capacity of a CSFV moderately virulent strain to induce trans-placental infection and congenital viral persistence, two pregnant sows (Sows 5 and 6) were inoculated with the Catalonia 01 (Cat01) strain. As in Experiment 1, the infection was carried out at 74 days of gestation, and a trained veterinarian recorded clinical signs daily.

The Cat01 infected sows did not show any clinical signs after inoculation. However, at 34 dpi (108 days of gestation), Sow 6 went into early labour and gave birth to eight stillbirths and two live piglets. All the stillbirths showed haemorrhagic lesions, whereas the live piglets were very weak and had to be euthanised on the same day for ethical reasons. The sow was also euthanised at this time. Both Cat01 infected sows were CSFV RNA positive in sera, and rectal and nasal swabs at 7dpi. The CSFV RNA load was low in all the samples, with Ct values ranging from 31 to 37. Afterwards, both sows cleared the virus, only Sow 6 was positive in rectal swab at 28 dpi, although at low RNA concentration (Ct 35.59) (data not shown).

### 2.10. Vertical Transmission and Congenital Viral Persistence Generated by the Moderate Virulence CSFV Strain 

At 114 days of gestation, Sow 5 gave birth to eight live piglets and six stillbirths, the live animals were active and fed normally from the mother immediately after birth. During the seven days after farrowing, two piglets were found dead in the pen, having being crushed by the sow, whereas no clinical signs were registered in the remaining six animals (Piglets 1 to 6). Piglets 1, 3, and 5 remained clinically healthy during the 32 days of the trial. Meanwhile, the other three piglets (Piglets 2, 4, and 6) developed sporadic fever peaks (below 41 ºC) from day 10 until the end of the study. Piglet 6 developed mild polyarthritis from day 10, and Piglets 2 and 4 at days 30 and 23, respectively. Notably, at the time of euthanasia, the piglets weighed around 8.5 kg and continued to show normal feeding behaviour. 

On the day of birth, the piglets were positive by RT-qPCR with high CSFV RNA load (Ct values about 23) in the rectal swab samples (Table 4). Despite the absence of CSF specific clinical signs, a high CSFV RNA load (Ct value about 20) was detected in all the serum samples during the study, indicating a permanent viremia in the piglets during the trial (Table 4). In parallel, high and permanent excretion in nasal and rectal swabs was found in all the sampling time points during the 32 days after birth. The Ct values increased in the majority of animals throughout the trial, reaching Ct values around 22 and 24 in nasal and rectal swabs (Table 4). 

### 2.11. Immune Response Generated by the Moderately CSFV Strain in Sows and Their Litters

**Figure 5:**
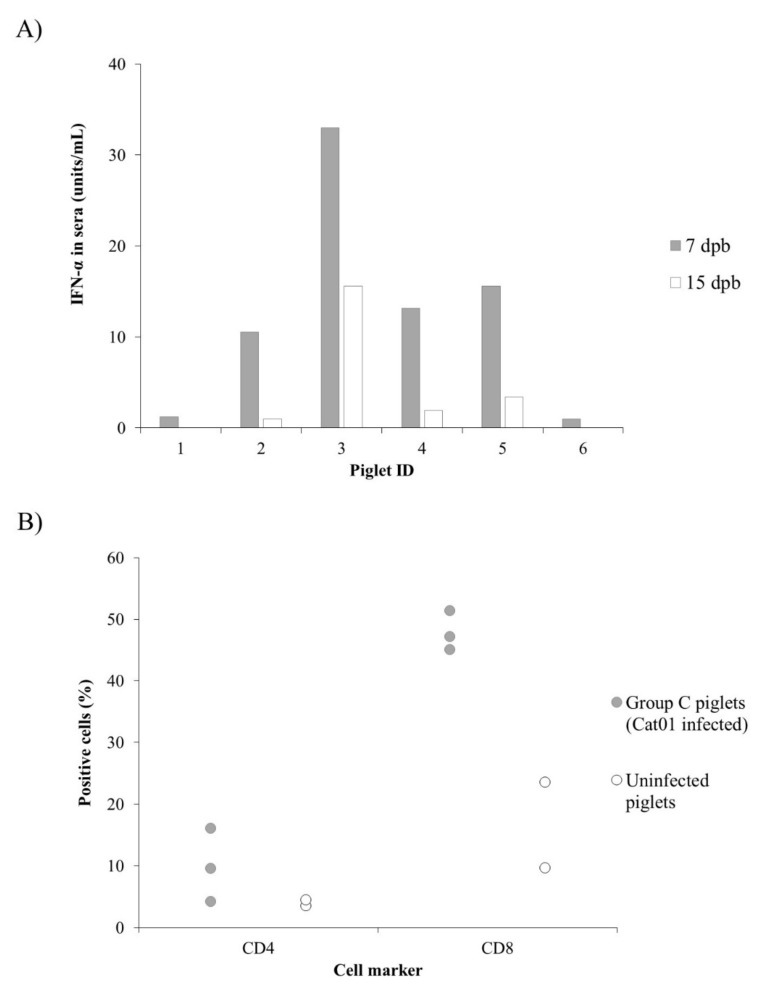
IFN-α levels and phenotypic profile in peripheral blood mononuclear cells (PBMCs) from CSFV congenital persistently infected piglets. (**A**) Concentration of IFN-α in sera expressed as units/mL from CSFV persistently infected piglets at 7 dpb (grey bars) and 15 dpb (white bars). (**B**) Comparative expression of the T-CD4+ and T-CD8+ cell subsets in PBMCs from uninfected piglets (white dots) and piglets infected with the Cat01strain (grey dots).

After infection, Sow 6 developed CSFV specific humoral response at 14 dpi, while Sow 5 was positive at 21 dpi, with blocking percentage values of 42% and 60%, respectively, which increased throughout the study. Neutralising antibody response appeared at 21 dpi on Sow 5 (titre 1:120), and at 14 dpi in Sow 6 (titre 1:20), and increased, reaching titres of 1:160 in both sows by 28 dpi. Nevertheless, none of the piglets showed an antibody response either by ELISA or NPLA during the 32 days after birth. Interestingly, IFN-α was detected in the sera from 4 piglets at 8 and 15 days post-birth (dpb) (Figure 5A). On the other hand, alterations in the CD4^+^ and CD8^+^ T-cell subsets from Cat01-infected piglets were found in the analysed PBMC from persistently infected piglets. While the T-CD4^+^ population did not exceed 5% in the uninfected, age-matched piglets, these cells ranged from 4% to 16% in persistently infected animals. On the other hand, the CD8^+^ cell subset was increased (about 50%) in the infected animals, being between 9.6 and 23.5% in the uninfected animals (Figure 5B).

## 3. Discussion

CSF congenital persistent infection was described several decades ago; however, some aspects regarding the generation of this form of the disease remain to be elucidated, and the available information is not up to date [4,6]. In the present work, three groups of sows were infected with either the Margarita, Cat01, or PdR CSFV strains. Each of these strains hav been previously characterised as of high, moderate, and low virulence, respectively [20,21,22]. In accordance with previous studies, the infection was carried out at 74 days of gestation, a time-point in which persistent congenital infection can be generated [4]. The capacity for trans-placental transmission and induction of foetal immune response was compared side by side between the high and low virulence strains (Figure 1 and Figure 2, Table 1 and Table 2). In accordance with previous data found in piglets, the highly virulent CSFV Margarita strain induced high serum IFN-α levels in sows over a short period of time [23]. By contrast, the IFN-α response induced by the low virulence PdR strain was lower, although it lasted one week longer. This supports the role of high replication rates for the previously described exacerbated innate immune response in the host after infection with highly virulent CSFV strains. This may explain the differences in pathogenesis between the sows from these two groups, with more severe lesions and an inability to clear the virus in the Margarita-infected sows, compared with the clinically healthy status and low replication of the PdR-infected ones. Trans-placental transmission was more efficient with the highly virulent Margarita strain, and high viral RNA load was detected in sera and tissues from the foetuses in this group. Conversely, a small proportion of the foetuses from the PdR infected sows were viraemic with high viral replication in organs, while the majority of them were either non-infected or only showed low viral RNA in tissues. Despite the immune response developed, mainly in Margarita infected sows, CSFV crossed the trans-placental barrier from the sows to their foetuses (Table 1 and Table 2, Figure 2). In agreement with previously described data, the high replication rate found in sows infected with a highly virulent CSFV strain may explain the activation of neutralising antibody response in these animals. However, taking into account that the onset of the antibody response in the sows was after two weeks, it is likely that the generation of trans-placental transmission took place during the first week after infection. Considering the previously described data, in order to avoid trans-placental transmission, it is necessary that effective neutralising antibody response be already present at the moment of infection, with titres of at least 1/320 [24].

Mummifications and haemorrhagic lesions were found in the Margarita infected foetuses. Probably, these animals would have died during the perinatal period. On the contrary, neither mummifications nor macroscopic lesions were observed in the PdR infected foetuses, even in those that showed viremia and high levels of viral replication in organs. It is very well known that sows transmit passive immunity to CSFV to the litters via colostrum [25,26]. These maternally derived antibodies (MDA) protect piglets against disease, including CSF, during their firsts weeks of life [15,25,26]. Considering that, in the case that the piglets had been born, the low immunity generated in the sows after infection with the low virulence PdR strain would result in an inefficient transmission of MDA to these litters. There might be major consequences to this situation since the suboptimal level of MDA would favour the infection of the non-infected piglets by their congenital persistently infected littermates and lead to chronic or postnatal persistent infection [10,11]. Recently, it was reported that the lack of maternal immunity led to a high prevalence of CSFV persistently infected piglets in an endemic scenario [10]. Notably, the CSFV persistently infected piglets have been proven to be refractory to vaccination [10,27]. This complex situation may lead to a vicious circle, which greatly impairs control programs of regions where CSF persistent infections are occurring.

In the case of infection carried out with the moderately virulent CSFV Cat01 strain, early labour in one of the infected sows and mummification and stillbirths in both of them were detected. Interestingly, both Cat01 infected sows developed a CSFV neutralising antibody response. However, the viral trans-placental transmission was not impaired, and all the piglets that were born alive in one of the Cat01 infected litters developed persistent congenital infection. These piglets showed normal weight gain, according to standards [28], despite being infected and excreting high viral load with a lack of CSFV specific antibody response [1,5,6]. Interestingly, the level of viral replication was comparable, or even higher than those found in the foetuses from sows infected with the high virulence strain (Table 2 and Table 4). This finding suggests an immunomodulatory capacity of the moderate virulence CSFV strains in the interaction with the host. Previous data showed the efficacy of this type of CSFV strain to also generate persistent postnatal infection [11,27]. Similar to persistent postnatal infection, low levels of IFN-α were found during congenital viral persistence, despite the high viral replication, pointing towards immunosuppressive regulation. Similar mechanisms might be taking place during the establishment of congenital or postnatal viral persistence. Recently, myeloid-derived suppressor cell populations have been determined to play a relevant role in the generation of CSFV postnatal persistence infection [29]. It cannot be discarded that these cell subsets are playing a role during the establishment of CSFV congenital persistent infection, considering that they have been found in cord blood and during neonatal stages in humans [30,31]. On the other hand, a low CD4/CD8 ratio has been reported as a marker for dysregulation of the immune response [32,33,34,35]. An increase in the CD8^+^ T-cell population, resulting in a low CD4/CD8 ratio, has been reported in CSFV postnatal persistently infected animals [12]. In the present study, an increase in the CD8^+^ T-cell subset was observed in the PBMC of infected foetuses and piglets from all the experimental groups. This finding may indicate that immunosuppressive mechanisms are also taking place in animals after trans-placental infection by CSFV. 

Activation of innate immunity, evidenced by the IFN-α and IFN-γ levels detected in sera, was found in the foetuses and piglets regardless of the infecting strain and the maturity level of the immune system (Table 3 and Figure 5). Type I interferon response activates the innate immunity after viral infection by playing an antiviral and immunomodulatory role. CSFV has the capacity to induce high levels of IFN-α response in pigs, being associated with disease severity and viral replication in the infected animals [36]. The highest IFN-α response was found in the viraemic foetuses or in those that showed higher viral replication in organs from the group infected with the low virulence PdR strain. Notably, the capacity of the PdR strain for high and prolonged IFN-α activation in piglets has been associated with an uninterrupted 36-uridine sequence found in the 3′ untranslated region of the CSFV genome [23]. Activation of IFN-α response in ruminant and human foetuses, following infection with bovine viral diarrhoea and Zika virus, respectively, has been described, and it may support the results obtained in this study [37,38]. Thus, the immunotolerance mechanism that was previously associated with the development of CSF congenital persistent form [1,5] is a complex immunologic phenomenon, and further studies may explain this mechanism and its relation with the establishment of viral persistence.

Previous reports have shown that the levels of sCD163 can be increased as a result of tissue damage during acute infection with highly pathogenic viruses, such as the African swine fever virus (ASFV) [39,40]. In addition, increased IFN-γ levels have also been found as part of the cytokine storm phenomenon responsible for the pathogenesis of ASFV [39,40,41]. In agreement with the haemorrhagic lesions and levels of viral replication found in foetuses infected with the high virulence CSFV Margarita strain, it is likely that the increase of IFN-γ and sCD163 may be associated with the exacerbated immune response in the host after infection, leading to cellular homeostasis imbalance and tissue damage. 

Taken together, our results show that the infecting CSFV strain capacity for viral replication influences its efficacy for trans-placental transmission and the establishment of persistent infection. Likewise, the CSFV strain with a moderate virulence degree proved to be very efficient in generating CSFV congenital persistent infection following trans-placental transmission. Our results indicate that trans-placental infection took place very fast before the neutralising antibody response could be generated in sows. Therefore, vaccines against CSFV indicated for pregnant sows must induce fast and strong immunity to guarantee the viral protection of their offspring against this type of infection.

On the other hand, the foetal immune system is able to recognise the virus and generate immune response after trans-placental infection. Further studies are needed to elucidate the mechanisms by which the specific immune response against CSFV is being impaired, following the initial recognition of the pathogen. To the best of our knowledge, this is the first report showing the foetal immune response after CSFV infection.

## 4. Materials and Methods

### 4.1. Cells and Viruses

Production of the viral strains was carried out by infecting susceptible cells with viral suspensions in 2% pestivirus-free foetal bovine serum using the porcine kidney cell line PK-15 (ATCC CCL 33, Middlesex, England), cultured in Eagle’s minimum essential medium supplemented with 5% foetal calf serum. Following the infection, cells were incubated at 37 ℃ in 5% CO2, and after 72 h, the virus was harvested. Peroxidase-linked assay (PLA) [42] was used for viral titration following the statistical methods described by Reed and Muench [43]. The CSFV PdR and the Margarita strains, both belonging to the 1.4 subgenotype [44,45], have been characterised as low and high virulence strains, respectively [20,21]. The Cat01 strain, which belongs to subgenotype 2.3, was selected as a moderate virulence prototype [22].

### 4.2. Experimental Design

Six pregnant sows (Landrace) of 68 days of gestation, from a commercial farm, were housed in the biosafety level 3 (BSL3) animal facility at CReSA (Barcelona, Spain). The animals were purchased from pestivirus-free farms, and they were also checked for antibodies against CSFV before arriving at the CReSA facilities. Animals were numbered from one to six and distributed in three groups (from A to C), each group in a separate box with standard facilities for pregnant sows. In accordance with the previously established methodology to evaluate the capacity of CSFV for trans-placental transmission, two sows were included in each experimental group [24,46]. After five days of acclimatisation period (74 days of gestation), Sows 1 and 2 (Group A) were inoculated with the CSFV Margarita strain, Sows 3 and 4 (Group B) with the PdR strain and Sows 5 and 6 (Group C) with the Cat01 strain. The viral dose for all the inocula was 10^5^ TCID _50_ per animal, and the inoculation was carried out by intramuscular injection in the neck [22,24,47]. After infection, a trained veterinarian recorded clinical signs daily in a blinded manner. Two experiments were carried out, Experiment 1 included Groups A and B, while Experiment 2 included the Group C sows.

In Experiment 1 of the trial, serum and nasal and rectal swab samples were collected on the day of infection and at 4, 8, 14, and 22 dpi, which corresponded with days 74, 78, 82, 88 and 96 of gestation, respectively. At this time, the sows were euthanised, following the accepted procedures accordingly with the European Directive 2010/63/EU. Whole blood in EDTA was obtained in the day of infection and before euthanasia for ex vivo collection of PBMCs. After necropsy, tissue samples from tonsil and Peyer’s patch were collected [24]. In parallel, the foetuses from all gilts were obtained, following procedures previously described to avoid foetal distress [24,48]. All foetuses were subjected to an exhaustive necropsy in which the presence of macroscopic lesions in different organs was evaluated [49]. Sera and whole blood samples and tissues (tonsil, spleen, and thymus) were collected from 13 foetuses per each sow.

In Experiment 2, sera samples were collected on the day of infection and at 7, 14, 21, and 28 dpi (days 81, 87, 95, and 102 of pregnancy, respectively). At farrowing, rectal swabs were collected from all piglets. Sows were kept with their litters for 21 days, and, after removal of the sow, piglets were fed an age-appropriate diet (StartRite, Cargill, Spain) until the end of the trial. The handling of the piglets was performed following previously described protocols [11].

Serum and nasal and rectal swabs were collected from piglets at 7, 15, 23, 27, and 32 dpb. At this time, whole blood samples were collected and piglets were euthanised following procedures according to the European Directive, using a pentobarbital overdose of 60–100 mg/kg of weight, administered via the jugular vein. In addition, sows and piglets were euthanised before the end of the trial if they presented clinical signs compatible with severe CSF or exhibited prostration behaviour, in accordance with previous studies [22]. The experiment was approved by the Ethics Committee for Animal Experiments of the Autonomous University of Barcelona (UAB), according to existing Spanish and European regulations. 

### 4.3. Detection of CSFV RNA 

The NucleoSpin RNA isolation kit (Macherey-Nagel, Düren, Germany) was used in order to extract RNA from sera and nasal and rectal swab samples, as well as from organ samples, following the protocol provided by the manufacturer. In all cases, a final volume of 50 μL of RNA was extracted from an initial sample volume of 150 µL. The detection of viral RNA was carried out by a previously described RT-qPCR assay [18], validated in our laboratory for the detection of CSFV RNA in sera, nasal, and rectal swabs and tissue samples [11,22]. Samples were considered positive when the Ct values were equal to or less than 42. In addition, using the Ct value, samples were determined to have either high (Ct value below 23), moderate (between 23 and 28), or low (Ct value above 28) CSFV RNA load, as previously described [23,50]. Samples in which fluorescence was undetectable (Undet) were considered negative.

### 4.4. Determination of E2-Specific and Neutralising Antibodies 

CSFV E2-specific antibodies were evaluated in sera from sows, foetuses, and piglets, using a commercial ELISA kit (IDEXX Laboratories, Liebfeld, Switzerland). Positive results were considered when the blocking percentage was ≥40%, following the manufacturer’s recommendations. Additionally, neutralising antibodies against the respective infecting strain were determined using an NPLA assay [19]; thus, animals from Groups A, B, and C were evaluated for neutralising antibodies against the Margarita, PdR, and Cat01 strain, respectively. The neutralising antibody titres were expressed as the reciprocal dilution of serum that neutralised 100 TCID of 50% of the culture replicates.

### 4.5. IFN-α ELISA Test in Serum Samples

IFN-α concentration was determined in sera from foetuses and sows from Groups A and B, as well as piglets from Group C at 7 and 15 dpb, using a previously described in-house ELISA test [11,51]. Briefly, plates were coated overnight with an anti-IFN-α monoclonal antibody (K9 clone, PBL Biomedical Laboratories, Piscataway, New Jersey, USA). After washing, 50 µl of serum samples and serial dilutions of IFN-α recombinant protein (PBL Biomedical Laboratories) were plated by duplicate and incubated for 1 hour at 37 ºC. Afterwards, plates were washed, and a biotinylated anti-IFN-α antibody was added (F17 clone, PBL Biomedical Laboratories). Following an incubation of 1 hour at 37 ◦C, the plates were washed, and streptavidin-HRP was added. Finally, after a 30 minute incubation, 3,3′,5,5′-tetramethylbenzidine (TMB) was used for revealing the technique, using H_2_SO_4_ 1N as a stop solution. Plates were read at 450 nm, and cytokine concentrations (units/ml) were determined using a regression line built with the optical densities of the cytokine standards used in the test.

### 4.6. ELISA Detection of IFN-γ and sCD163

IFN-γ and sCD163 were analysed in sera from foetuses and sows from Groups A and B. Commercial ELISA test was used for detection of IFN-γ (IFN-γ ELISA Kit, Porcine, Life Technologies), following the manufacturer’s instructions and the results were expressed as picograms per millilitre (pg/ml). Finally, a formerly described ELISA using lysates from CD163 transfected CHO cells as standard was used to quantify sCD163 [39,52]. Results were expressed as the equivalent numbers of CD163-transfected CHO cells (ENC). 

### 4.7. PBMCs Collection and Flow Cytometry Assay

PBMCs were obtained from whole blood collected at the time of necropsy from three animals of each group in Experiments 1 and 2 of the trial, previously characterised by RT-qPCR. Cells were separated by density-gradient centrifugation with Histopaque 1077 (Sigma-Aldrich St. Louis, MO, USA), followed by osmotic shock in order to eliminate the remaining red blood cells. The number and viability of the PBMCs were determined by staining with Trypan Blue [21]. Additionally, thymocytes were obtained from three uninfected foetuses, and whole blood samples were also collected from three uninfected foetuses and piglets at the same time of gestation/days after birth as the foetuses from Experiment 1 or the piglets from Experiment 2, respectively. 

The phenotypic profile of PBMCs from foetuses and piglets was evaluated by flow cytometry. Single staining was performed using the mAbs to porcine CD4 (74-12-4, IgG2b) Alexa Fluor 647 conjugate (BD Biosciences), and CD8-α (76-2-11, IgG2a) FITC-labelled (BD Biosciences, Franklin Lakes, NJ, USA). 

The staining protocols were performed as previously described [11,12]. After staining, cells were filtered and passed in the cytometer (FACSAria IIu, BD Biosciences), with 10,000 cell events being recorded for each sample. The cells were analysed by FACSDiva software, version 6.1.2 and the results were expressed as the percentage of positive cells obtained for each staining, using irrelevant isotype-matched mAbs as staining controls.

## Figures and Tables

**Table 1:** Neutralising antibody response in sows after CSFV infection.

		Time after CSFV Infection			
Sow ID		0 dpi	8 dpi	14 dpi	22 dpi
Margarita infection group	1	0	0	1:640	1:1280 *
	2	0	0	1:320	1:960
PdR infection group	3	0	0	0	1:60
	4	0	0	0	0

* sample taken at 17 dpi.

**Table 2:** Detection of CSFV RNA in foetuses from sows infected with CSFV Margarita or PdR strains.

Margarita Infection Group A	CSFV RT-qPCR (Ct Value)				PdR Infection Group B	CSFV RT-qPCR (Ct Value)			
Foetus ID	Serum	Tonsil	Spleen	Thymus	Foetus ID	Serum	Tonsil	Spleen	Thymus
*Foetus from Sow 1*					*Foetus from Sow 3*				
1	19.65	18.52	17.91	16.54	1	Undet.	Undet.	34.65	Undet.
2	23.13	20.61	19.56	25.86	2	Undet.	Undet.	Undet.	Undet.
3	20.40	21.96	19.11	15.70	3	Undet.	32.73	29.94	35.45
4	21.35	26.58	18.95	16.85	4	Undet.	Undet.	32.44	Undet.
5	18.48	19.69	17.00	19.18	5	Undet.	Undet.	37.43	Undet.
6	17.65	19.59	16.14	15.92	6	Undet.	35.32	33.82	Undet.
7	18.71	20.53	16.59	16.46	7	22.42	22.75	15.86	17.69
8	23.23	19.92	17.75	15.56	8	Undet.	36.67	Undet.	Undet.
9	18.73	16.13	16.24	14.70	9	Undet.	Undet.	Undet.	Undet.
10	19.55	18.78	16.85	17.31	10	Undet.	36.60	34.56	Undet.
11	20.56	23.52	18.45	17.18	11	24.76	23.18	18.30	21.15
12	17.58	18.05	16.62	16.31	12	32.36	23.37	23.68	24.85
13	17.27	18.61	16.62	16.37	13	Undet.	33.91	31.51	36.33
**Mean**	**19.72**	**20.33**	**17.49**	**17.28**	**Mean**	**38.50**	**37.19**	**33.28**	**36.33**
**Desvest**	**2.05**	**2.72**	**1.22**	**2.92**	**Desvest**	**7.16**	**5.88**	**9.89**	**9.02**
*Foetus from Sow 2*					*Foetus from Sow 4*				
1	20.30	25.16	17.84	20.28	1	Undet.	Undet.	Undet.	Undet.
2	18.68	17.92	17.44	15.79	2	28.26	28.36	18.37	21.79
3	18.50	19.28	17.43	16.95	3	Undet.	35.01	28.32	28.24
4	27.06	28.74	25.01	24.94	4	Undet.	34.59	36.29	Undet.
5	18.94	16.87	17.36	15.70	5	Undet.	Undet.	Undet.	36.54
6	19.51	18.93	17.44	16.78	6	Undet.	Undet.	Undet.	Undet.
7	16.81	18.01	16.93	16.78	7	Undet.	Undet.	Undet.	Undet.
8	16.60	17.25	23.57	17.51	8	Undet.	Undet.	Undet.	36.58
9	18.65	18.36	16.47	16.85	9	Undet.	Undet.	Undet.	Undet.
10	20.67	22.67	17.22	16.97	10	Undet.	Undet.	34.36	Undet.
11	16.91	18.37	17.26	16.92	11	34.54	29.63	26.51	25.84
12	16.94	18.37	18.96	17.66	12	Undet.	36.52	Undet.	Undet.
13	15.67	16.88	16.41	16.26	13	21.52	20.59	18.58	18.32
**Mean**	**18.75**	**19.30**	**18.46**	**17.43**	**Mean**	**38.87**	**36.88**	**35.16**	**36.70**
**Desvest**	**2.99**	**3.34**	**2.81**	**2.44**	**Desvest**	**6.69**	**7.01**	**9.21**	**8.55**

Undet: undetectable, negative sample.

**Table 3:** IFN-α levels in foetal serum.

Margarita Infection Group A		PdR Infection Group B	
Foetus ID	IFN-α	Foetus ID	IFN-α
*Foetus from Sow 1*		*Foetus from Sow 3*	
1	**48.5**	1	0.0
2	0.0	2	0.0
3	0.0	3	**60.3**
4	**237.7**	4	0.0
5	0.0	5	0.0
6	**18.7**	6	0.0
7	0.0	7	**116.7**
8	0.0	8	0.0
9	0.0	9	0.0
10	**31.3**	10	0.0
11	0.0	11	**102.7**
12	**8.0**	12	**209.6**
13	0.0	13	0.0
*Foetus from Sow 2*		*Foetus from Sow 4*	
1	0.0	1	0.0
2	**25.5**	2	**227.8**
3	**26.7**	3	**239.5**
4	**49.3**	4	0.0
5	0.0	5	0.0
6	0.0	6	0.0
7	0.0	7	0.0
8	**14.3**	8	0.0
9	0.0	9	0.0
10	**12.2**	10	0.0
11	**43.2**	11	**173.4**
12	0.0	12	0.0
13	0.0	13	**126.9**

**Table 4:** Detection of CSFV RNA in piglets from Sow 5, infected with the CSFV Cat01 strain.

CSFV qRT-PCR (Ct Value)																
	Day of Birth	7 dpb			15 dpb			23 dpb			27 dpb			32 dpb		
Piglet ID	Rectal Swab	Serum	Nasal Swab	Rectal Swab	Serum	Nasal Swab	Rectal Swab	Serum	Nasal Swab	Rectal Swab	Serum	Nasal Swab	Rectal Swab	Serum	Nasal Swab	Rectal Swab
1	25	17.06	24.77	23.77	16.88	16.87	24.52	16.55	23.03	22.38	16.51	24.70	23.62	17.30	18.80	22.47
2	23.94	16.10	26.09	20.42	16.34	25.76	23.91	16.06	20.82	25.53	15.96	21.16	23.52	17.28	20.59	27.20
3	24.01	16.01	23.15	21.46	16.36	25.88	23.36	16.80	27.16	24.49	17.29	21.66	23.74	19.27	19.25	24.42
4	22.63	16.31	22.30	20.86	16.74	19.26	26.91	17.01	18.02	25.36	16.73	22.20	25.44	16.36	23.53	22.86
5	23.44	15.71	27.94	22.81	16.68	27.00	24.38	16.61	20.42	24.77	16.89	20.41	25.83	17.64	19.30	25.99
6	24.55	15.39	23.20	23.79	25.52	22.65	26.56	16.56	21.28	23.19	16.66	20.57	22.70	16.74	19.86	>22.42
**Mean**	**23.93**	**16.10**	**24.58**	**22.19**	**18.09**	**22.90**	**24.94**	**16.60**	**21.79**	**24.29**	**16.67**	**21.78**	**24.14**	**17.43**	**20.22**	**24.23**
**Desvest**	**0.83**	**0.57**	**2.13**	**1.47**	**3.65**	**4.09**	**1.45**	**0.32**	**3.09**	**1.25**	**0.44**	**1.58**	**1.22**	**1.01**	**1.73**	**2.01**

dpb: days post birth.
